# A Naso-Orbito-Ethmoid (NOE) Fracture Associated with Bilateral Anterior and Posterior Frontal Sinus Wall Fractures Caused by a Horse Kick—Case Report and Short Literature Review

**DOI:** 10.3390/medicina55110731

**Published:** 2019-11-09

**Authors:** Florin Onișor-Gligor, Paul Andrei Țenț, Simion Bran, Mihai Juncar

**Affiliations:** 1 Department of Oral and Maxillofacial Surgery, “Iuliu Hatieganu” University of Medicine and Pharmacy, Strada Victor Babeș 8, 40000 Cluj-Napoca, Romania; dr_onisorf@yahoo.com (F.O.-G.); dr_brans@yahoo.com (S.B.); 2 Department of Oral and Maxillofacial Surgery, University of Oradea, 10, Piața 1 Decembrie Street, 410073 Oradea, Bihor, Romania; mihaijuncar@gmail.com

**Keywords:** naso-orbito-ethmoid (NOE) fracture, maxillofacial, trauma, treatment, open reduction, frontal sinus

## Abstract

Naso-orbito-ethmoid (NOE) fractures associated with anterior and posterior frontal sinus wall fractures are among the most challenging cranio-maxillofacial injuries. These represent a major emergency, having a potentially severe clinical picture, with intracranial hemorrhage, cerebrospinal fluid (CSF) leak, meningeal lesions, pneumocephalus, contusion or laceration of the brain matter, coma, and in some cases death. In this article, we present the case of a 30-year-old patient with the diagnosis of NOE fracture associated with bilateral anterior and posterior frontal sinus wall fractures caused by a horse kick, with a fulminant post-traumatic alteration of the neurological status and major impairment of the midface bone architecture. Despite the severity and complexity of the case, early initiation of correct treatment both in terms of intensive care and cranio-maxillofacial surgery led to the successful rehabilitation of the neurological status, as well as to the reconstruction and redimensioning of midface architecture and, not least, to the restoration of the patient’s physiognomy.

## 1. Introduction

A person’s physiognomy is directly dependent on the bone architecture and overlying soft tissues of the midface [1]. Naso-orbito-ethmoid (NOE) fractures alter the shape and projection of the nose, orbital contour, palpebral contour, and implicitly the eye shape, inducing severe functional and cosmetic disorders that practically disfigure the patient [2]. A key anatomical element that is affected in the case of NOE fractures is the medial canthal tendon (MCT), which inserts by two fascicles: an anterior fascicle in the anterior lacrimal crest of the maxillary frontal process and a posterior fascicle in the posterior lacrimal crest of the lacrimal bone, with the lacrimal sac between the two crests [3]. MCT represents the medial insertion of the eyelids, but it also plays a role in lacrimal sac drainage [4]. The alteration of the MCT insertion has severe physiognomic consequences, clinically manifesting by rounded palpebral fissures and telecanthus [5]. Markowitz and Manson [6] classify NOE fractures into three types, depending on the maintenance of the MCT insertion in the fractured bone fragments: Type I, when MCT insertion is intact in a single large bone fragment, type II, when MCT insertion is attached to a comminuted bone fragment, and type III, when MCT is avulsed from the medial orbital wall. The correct diagnosis of NOE fractures can be obtained through a competent combination of clinical and imaging examination [6]. Clinical examination based on inspection and palpation is often difficult to perform due to post-traumatic edema, drastic pain, and potential soft tissue associated injuries, which can mask the exact characteristics of the underlying fractures; under these conditions, computed tomography with three-dimensional reconstruction is mandatory to sustain the clinical diagnosis [7]. The treatment of these fractures is complex and aims to reconstruct the midface skeleton, to anatomically reinsert the MCT, and to restore the symmetry of intercanthal soft tissues [7]. Due to the diversity of the clinical picture at presentation, the absence of early diagnosis, and potential long-term sequelae, the treatment of NOE fractures is still controversial in the literature [3]. The association of a NOE fracture with frontal sinus wall fractures exponentially increases the complexity and morbidity of the case regarding both local and general treatment [8]. When frontal bone fractures are associated, the possibility of posterior frontal sinus wall involvement and the integrity of the nasofrontal canal should be evaluated [9]. Posterior wall fractures may present a severe clinical picture with intracranial hemorrhage, cerebrospinal fluid (CSF) leak, meningeal lesions, pneumocephalus, contusion or laceration of the brain matter with a fulminant alteration of the neurological status, coma, or even death [9]. The treatment of these fractures is difficult and challenging, and most frequently requires the multidisciplinary collaboration of a neurosurgeon, an oral and maxillofacial surgeon, and an intensive care specialist [10]. The therapeutic approach is aimed at reconstructing the bone and soft tissue architecture, as well as at preventing long-term complications such as mucocele, encephalocele, osteomyelitis, chronic sinusitis, meningitis, brain abscess, or even death [9].

In what follows, we present the complex and rare case of a 30-year-old patient with a type II naso-orbito-ethmoid (NOE) fracture associated with bilateral anterior and posterior frontal sinus wall fractures caused by a horse kick. The objectives of this article are to emphasize once more the major importance of early surgery in NOE and frontal sinus fractures in order to obtain a cosmetic, aesthetic and functional outcome, as well as to review the literature with the aim of choosing the ideal treatment depending on the patient’s fracture pattern, clinical picture, and associated lesions.

## 2. Case Report

A patient of 30 years old presented to the territorial emergency service. Due to progressive alteration of the neurological status, the patient was intubated in emergency and transferred in a state of induced coma to the service of Anesthesia and Intensive Care, Cluj-Napoca.

Clinical extraoral examination showed asymmetry of the upper face, the patient having a larger interorbital distance, telecanthus, slightly rounded palpebral fissures, major intrusion and flattening of the nasal bones, bilateral periorbital hematoma, bilateral bulbar conjunctival chemosis, and massive bilateral periorbital, centrofacial, and frontal edema, as well as two contused wounds in the right supraorbital and nasal pyramid region, which were sutured in separate points in the territorial service. Because the patient was in induced coma, functional ocular tests could not be performed at the time of examination. On palpation, bone discontinuity in the left inferior orbital margin, a comminuted fracture with depression of the nasal pyramid with major bone fragment mobility, loss of bone projection with depression of the medial orbital walls, the glabellar region, and the squamous part of the frontal bone were detected. Also, palpation of the skin in the frontal and glabellar region evidenced crepitations suggestive of subcutaneous emphysema. In order to assess the maintenance of the MCT insertion, a bowstring test was performed bilaterally, confirming the bilateral preservation of MCT insertions in the fractured bone fragments, which was classified as a type II NOE fracture according to Manson and Markowitz. The patient had no clinical signs suggesting a cerebrospinal fluid (CSF) fistula or epiphora.

**Figure 1:**
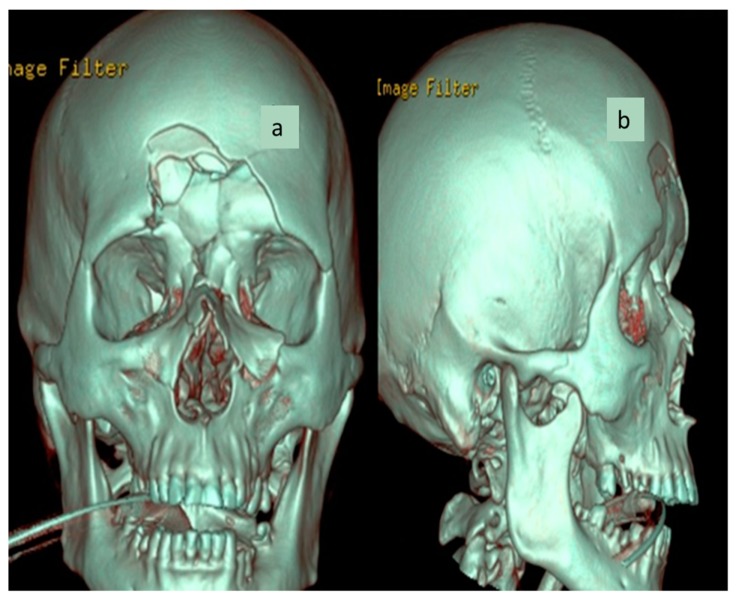
(**a**) Three-dimensional CT reconstruction—coronal. (**b**) Three-dimensional CT reconstruction—sagittal.

**Figure 2:**
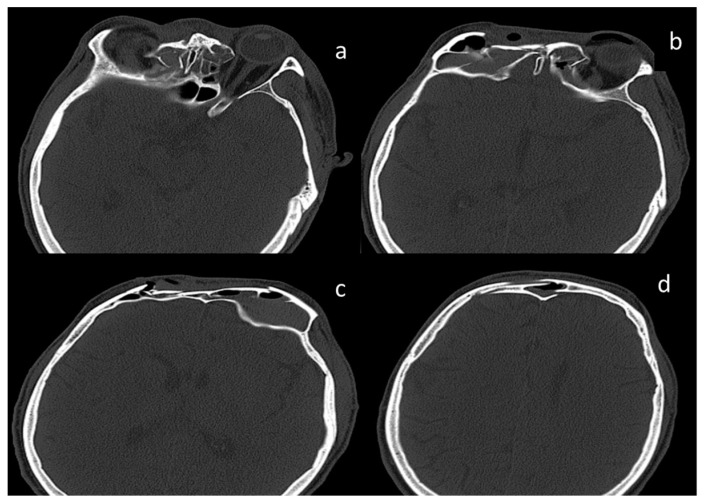
CT images, bone window, axial sections. (**a**) Naso-orbito-ethmoid (NOE) fracture with major depression and ethmoid hemosinus, (**b**,**c**) comminuted fracture of the anterior frontal sinus walls with frontal hemosinus, subcutaneous emphysema, and left pneumo-orbitis, (**d**) displaced posterior frontal sinus wall fracture.

**Figure 3:**
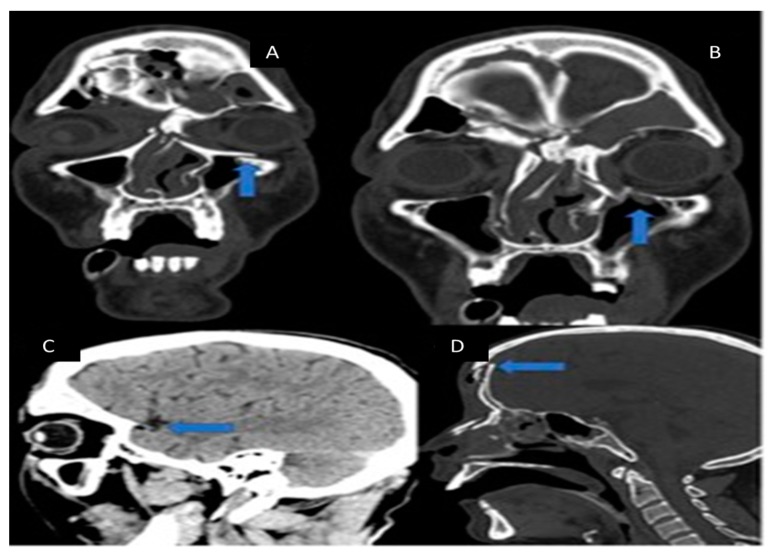
(**A**) CT image, coronal section—a telescoping fracture of the left inferior margin can be observed; (**B**) right inferior muscle incarceration in the fracture focus; (**C**) CT image, sagittal section—minimal pneumocephalus; (**D**) displaced posterior frontal sinus wall fracture.

Three-dimansional CT reconstruction of the viscerocranium (Figure 1a,b) evidenced a NOE fracture (comminuted fracture of the nasal bones and medial orbital walls with major depression with altered orbital volume bilaterally), a left inferior orbital margin fracture with displacement and right inferior muscle incarceration, a comminuted fracture with depression of the bilateral anterior frontal sinus wall and the outer cortex of the squama of the frontal bone, a posterior frontal sinus wall fracture with minimal displacement, bilateral ethmoid hemosinus, bilateral frontal hemosinus, minimal pneumocephalus in the median frontal lobe region, moderate subcutaneous emphysema of the frontal and glabellar region, as well as left intraorbital emphysema. No intra- or extraneuraxial, infra- or supratentorial blood accumulation and no signs of brain matter contusion were detected (Figure 2 and Figure 3).

After three days of Intensive Care Department monitoring, the patient was brought out of the induced coma and transferred into our service for reconstructive surgery.

**Figure 4:**
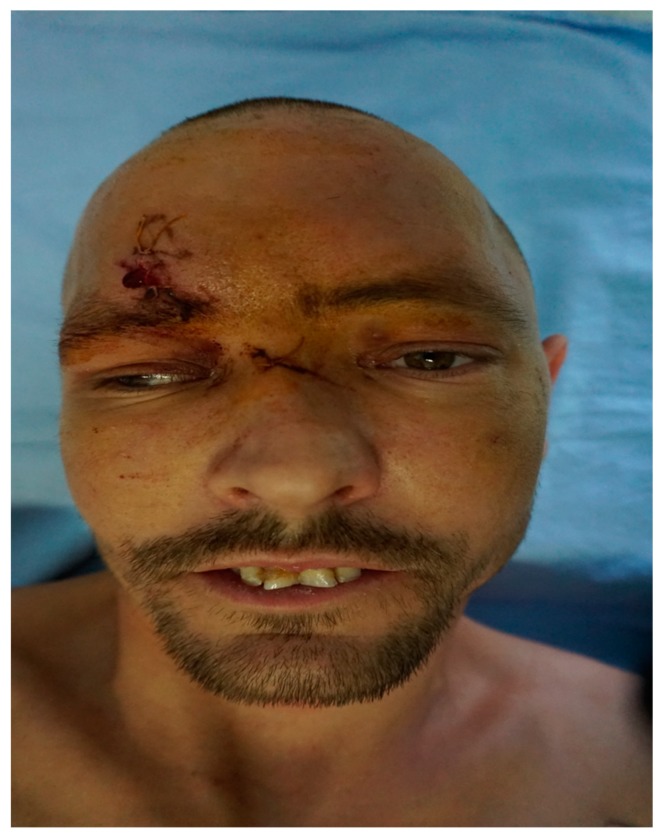
Preoperative appearance—hypertelorism with canthal dystopia, flattening of the nasal bones and impossible left eye globe movement due to right inferior muscle incarceration are seen.

At the time of the transfer, the patient complained of hyposmia and diplopia when looking forward, downward, laterally, and medially; the considerable limitation of the left eye globe movement in the mentioned directions being also clinically evidenced (Figure 4).

**Figure 5:**
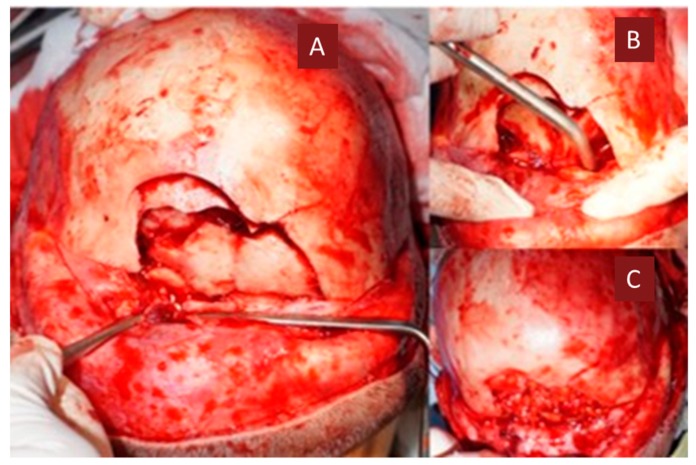
(**A**) Initial intraoperative appearance with the evidencing of the frontal fracture focus, (**B**) intraoperative appearance after bilateral radical frontal sinus surgery, (**C**) intraoperative appearance—obliteration of the frontal sinuses with abdominal cellular adipose tissue.

Surgery was carried out under general anesthesia with orotracheal intubation. A bicoronal approach with subgaleal dissection was used. In the frontal fracture foci, dissection of adhesions and incarceration of the epicranial aponeurosis, as well as of the frontal portion of the fronto-occipital muscle, was required (Figure 5A). The fractured fragments belonging to the outer cortex of the frontal bone and anterior frontal sinus walls were removed and kept in gentamicin solution, 80 mg/2 mL. Radical surgery of both frontal sinuses was performed (Figure 5B).

With the posterior frontal sinus wall being minimally displaced, without signs of dura mater herniation and without signs of cerebrospinal fluid leak, no surgical manipulation was required. The NOE fracture was reduced by anterior traction with Walsham forceps. The maintenance of the adhesion of the fractured fragments of the medial orbital walls to the periosteum, as well as the maintenance of the bilateral MCT insertion, facilitated the closed reduction of the fractured fragments at this level, without the need for reconstruction of the medial orbital walls with titanium meshes. The nasal bones were reduced and immobilized bilaterally with an osteosynthesis plate to the fixed portion of the supraorbital margins. The nasal septum was repositioned on the median line and immobilized using two internal conformers of sterile iodoform gauze. Furthermore, an external conformer for the protection of the reduced nasal bones was adapted and fixed with sterile dressing to the skin.

**Figure 6:**
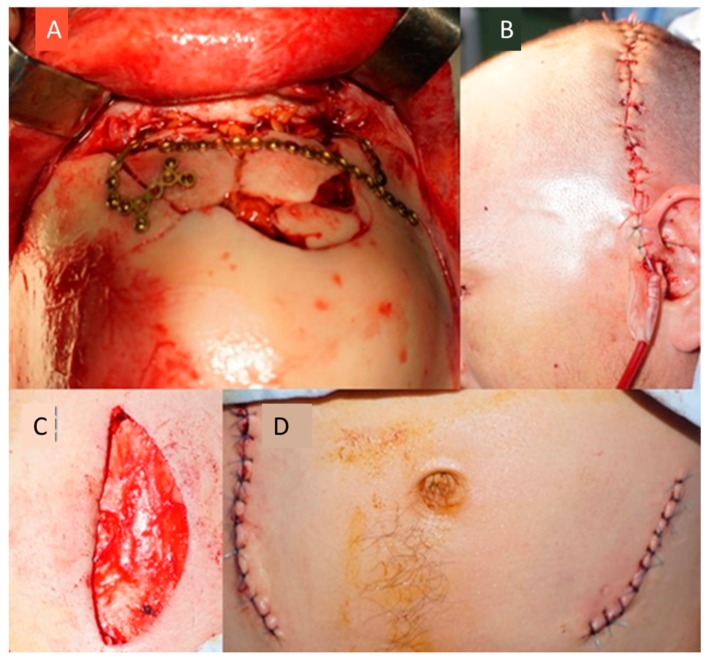
(**A**) Final intraoperative appearance with reconstruction of the bilateral frontal structure; (**B**) immediate postoperative appearance; (**C**,**D**) appearance after collection of abdominal cellular adipose tissue.

The frontal sinuses were obliterated with cellular adipose tissue taken from both abdominal flanks. The fractured fragments of the outer cortex of the frontal bone squama and the anterior walls of the two frontal sinuses were anatomically repositioned and fixed by osteosynthesis with miniplates and screws. Wound drainage was performed by positioning two drain tubes in the subgaleal region, which were extruded in the preauricular area. The bicoronal wound was sutured in two planes with sutures at separate points (Figure 5C and Figure 6).

**Figure 7:**
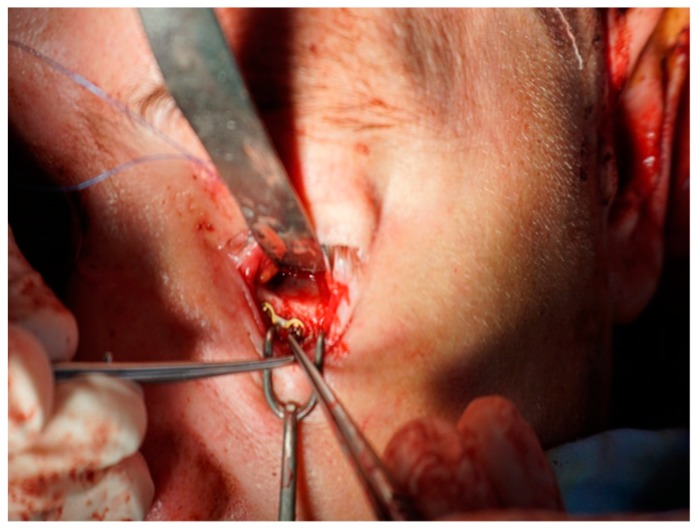
Intraoperative appearance—open reduction of the left inferior orbital margin fracture by infraciliary palpebral approach and internal fixation by osteosynthesis.

The left inferior orbital margin fracture was openly reduced and fixed with an osteosynthesis miniplate and six screws by infraciliary palpebral approach, after decarceration of the right inferior muscle and left periorbital fat was performed. The infraciliary skin wound was sutured with a 6.0 continuous intradermal polypropylene suture (Figure 7).

Postoperatively, the patient received daily meropenem, 1000 mg 3 × 1; cefort, 2 × 1 g; vancomycin, 2 × 1 g; dexamethasone, 2 × 8 mg/2 mL; tramadol, 150 mg 3; Ringer solution, 500 mL 3 × 1 fl; NaCl 0.9%, 100 mL-9 mg/mL 3 × 1 fl; Kabiven, 1 fl; vitamin C, 2 × 750 mg/5 mL; Betabioptal, 0.2 g + 0.5 g/100 mL ophthalmic drops, 3 × 3 drops in the conjunctival sac bilaterally.

**Figure 8:**
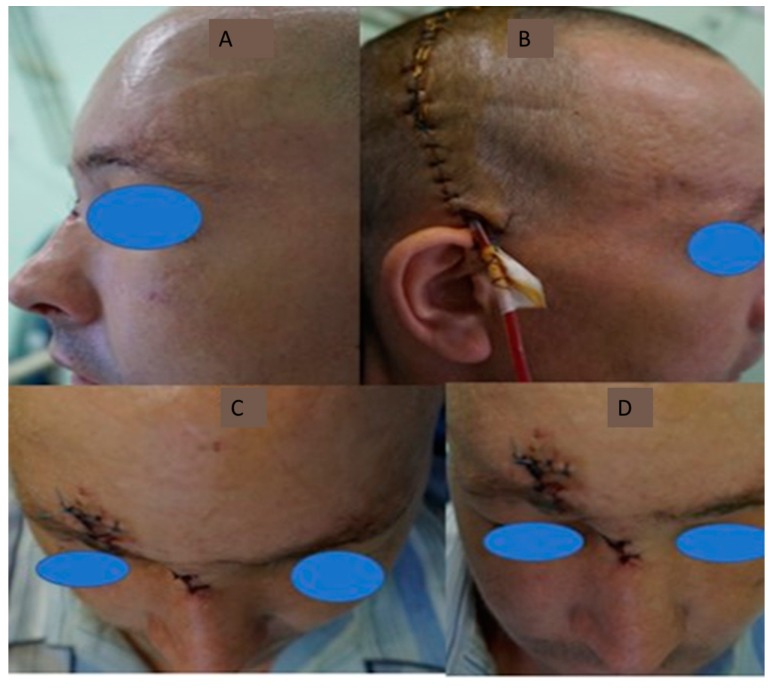
Appearance on the seventh postoperative day. (**A**) clinical sagittal facial aspect - the restoration of the nasal projection and the glabular contour; (**B**) Aspect of the bicoronal scar right before suture thread suppression; (**C**,**D**) We notice the symmetry of the nasal bones, as well as the restoration of the intercantal distance.

The patient’s evolution was favorable, with the restoration of left eye globe mobility within normal limits and complete disappearance of diplopia immediately postoperatively. On the fifth postoperative day, the intranasal and extraoral conformers were removed. Clinical examination showed symmetrical MCT insertion, restoration of intercanthal distance and bilateral symmetry of the mid-pupillary lines in relation to the lateral and medial canthal ligaments, restoration of the anatomical shape of the palpebral fissures, restoration of nasal projection and nasal contour, and restoration of the frontal squamous portion and glabellar region (Figure 8).

The patient was discharged on the eighth postoperative day with a medical prescription for Betabioptal, 0.2 g + 0.5 g/100 mL ophthalmic drops, 3 × 3 drops in the conjunctival sac for another five days.

**Figure 9:**
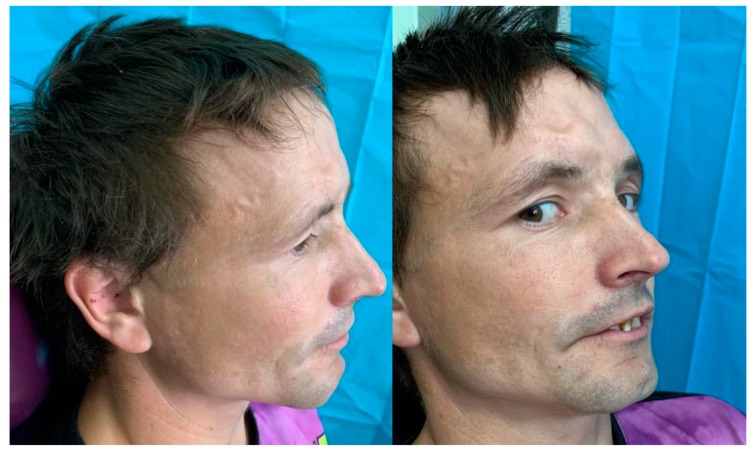
Clinical aspect nine months after surgery.

**Figure 10:**
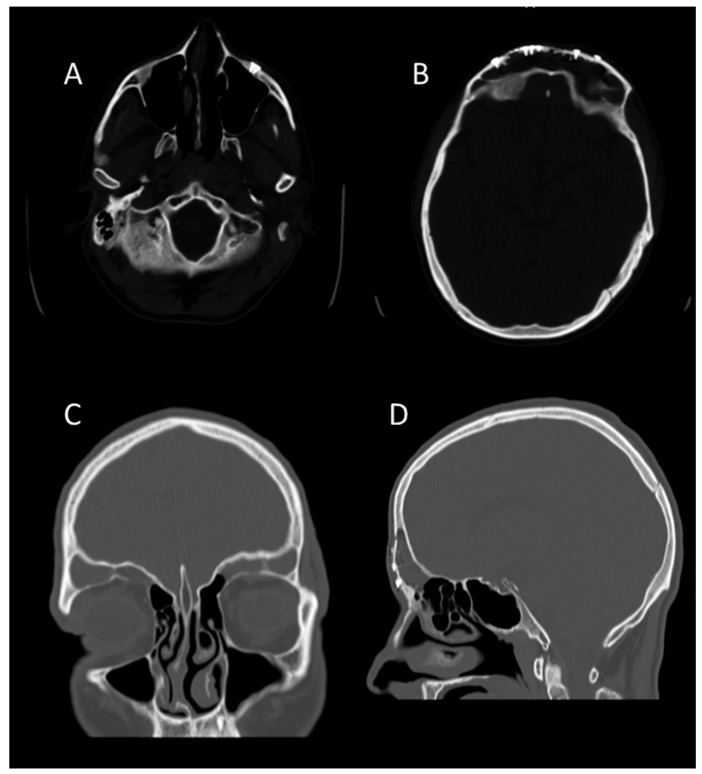
CT-scan aspect months after surgery. (**A**) the bone consolidation at the level of the lower left orbital rim and the symmetry of the bone contours at this level are highlighted; (**B**) bone consolidation with anatomical restoration of the anterior walls of the frontal sinuses; (**C**) coronal section that highlights the symmetry of the orbital dimensions, the symmetry of the frontal and ethmoidal sinuses contours; (**D**) sagittal section that highlights the anatomical position bone consolidation at the glabellar and nasal level.

After hospital discharge, the patient presented for monthly checks to assess the maintenance of aesthetic appearance and also sinusal and ocular functionality. Nine month after surgery the patient has a clinically unchanged appearance with the maintenance of the frontal bone contour, glabellar contour and of nasal bones projection, with the maintenance of the ocular mobility in normal parameters, without any functional disorders (Figure 9). On this occasion, we carried out a control computed tomography scan to evaluate the bone consolidation in the fracture foci (Figure 10). 

## 3. Discussion

Although NOE fractures are rare, their frequency is currently increasing due to the rise in the incidence of high kinetic energy trauma [3,11,12]. The most common causes of NOE fractures are motor vehicle collisions, followed by road traffic accidents and interpersonal violence, while fractures caused by horse kicks are extremely rare in the literature [11,12,13,14]. Due to the action of a high kinetic energy causative agent, NOE fractures are often associated with other fracture foci in the frontal bone, orbital walls, or midface [15,16,17]. In the present case, the injury caused by a horse kick was severe, inducing associated displaced comminuted fractures in the anterior and posterior walls of both frontal sinuses. Despite the fracturing of the posterior frontal sinus wall and the inner cortex of the frontal bone squama, the kick was not sufficiently strong to cause brain laceration, intracranial hemorrhage, cerebrospinal fluid (CSF) leak, or cerebral contusion, lesions that may accompany the fractures located at this level. The patient only had minimal pneumocephalus [1,2,3,4,5,6,7,8,9,10]. The clinical evaluation and the preoperative imaging must determine the pattern of fractures, the status of the medial cantus insertion, the possible leakage of cerebrospinal fluid, and the involvement of the nasolacrimal canal [7,8]. The maintenance of the insertions of the medial cantus can be palpably assessed through a bow string test [9]. However, marked edema often prevents this, leading to inconclusive results [10]. Cerebrospinal fluid loss may be clinically present as “runny nose” (straw-colored or clear nasal drainage) [11,12]. It can be assessed through various diagnostic methods: halo sign (not specific), the evaluation of glucose concentration between the nasal fluid and patient’s serum, or the beta—transferrin laboraty test [18,19,20]. The presence of base skull fracture lines on the CT-scan completes the diagnosis [18,19,20]. The impairment of the nasolacrimal duct is clinically manifested by the epiphora, however, three-dimensional reconstruction CT scan with axial, coronal, and sagittal sections is mandatory for final diagnosis [18,19,20,21]. Although the posterior cortex of the maxillary sinuses was minimally displaced, there was a risk of meningeal contamination with germs from both the frontal sinuses and the skin because of the supraorbital wound that opened the frontal fracture focus before suturing. Standard prophylaxis of meningitis in cranio-maxillofacial trauma is obtained by intravenous administration of third class cephalosporins (ceftriaxone 2 × 1 g/day) in association of vancomycin, due to the global increase in bacterial resistance to ceftriaxone nowadays [18,19,20,21].

The treatment of NOE fractures is controversial, difficult, and varies depending on the type of fracture and the associated fracture lines [1,2,3,4,5,6,7,8,9,10,11,12,13,14,15,16]. Type I NOE fractures are treated by closed methods in the case of fragment stability after reduction, while type II and III fractures or NOE fractures associated with frontal bone fractures require open exposure of the operative field, with direct reduction and osteosynthesis fixation of the fractured fragments, obliteration or cranialization of the frontal sinuses, and in the case of MCT avulsion, the association of intraoperative canthopexy is mandatory [3,7,21,22,23]. In the present case, we chose the extraoral bicoronal approach, which is currently considered the gold standard in the treatment of frontal sinus and naso-orbito-ethmoid (FSNOE) fractures [3,4,5,6,7,8,21,22,23,24]. The advantages of this flap are: a good exposure of the entire squamous portion of the frontal bone, bilateral frontal sinus walls, supraorbital margins, nasal bones, and bilateral medial orbital walls, the possibility of concomitantly taking bone grafts from the calvaria when reconstruction is needed, and the masking of scars in the hairy scalp skin [3,4,5,6,7,8,21,22,23,24]. However, some authors indicate a number of potential postoperative complications following this type of approach, and recommend avoiding bicoronal incisions whenever possible [25,26,27,28]. These complications include postoperative scalp hypomobility, alopecia of the bicoronal scar, partial or total paralysis of the frontal muscle, subgaleal hematoma, and the fact that they are time-consuming [25,26,27,28,29,30,31,32,33,34].

After the NOE complex reduction we did not attempt to perform safety canthopexy because it would have involved the risk of additional lacrimal bone fragmentation as a result of drilling and the subsequent risk of destabilization of MCT insertions [4,6,12,35]. In the contrary case, medial orbital wall reconstruction and canthopexy are required [1,2,3,4,5,6,23]. In terms of sequencing, bone contours will always be reconstructed first [1,2,3,4,5,6,7,8,9,10,11]. This can be achieved using preformed titanium meshes, autologous bone grafts (calvaria, mandible, iliac crest, tibia, etc.) or combinations of these, depending on the pattern of the post-traumatic defect [1,2,3,4,5,6,7,8,9,10,11,12,13,14,15,16,17]. Canthopexy can be performed by many methods, transnasal wiring currently being the method of choice [1,2,3,4,5,6]. Other methods described are canthopexy using stainless steel screws [36], transcaruncular barb and miniplate [37], cantilevered miniplate [38] or the combined transcaruncular-transnasal suture technique [39]. We successfully reconstructed nasal projection and contour by fixing the nasal bones to the supraorbital margins left intact using osteosynthesis with miniplates and screws; otherwise, nasal bone grafting would have been required [4,36,37,38,39,40]. Even though the nasal bones were rigidly fixed to the cranium, an intranasal conformer was necessary for five days in order to prevent the medial displacement of the frontal process of the maxilla and lacrimal bones [1,2,3,4,5,6]. Nevertheless, in the case of defective manipulation of the intranasal conformer, there is a risk for the fracture foci to heal in a hypercorrection position in the medial orbital walls, which clinically manifests by the persistence of telecanthus, hypertelorism, and inaesthetic nasal projection, resulting in major surgical failure [2,41,42,43].

The treatment of frontal sinus wall fractures is controversial and its aim is, in addition to the three-dimensional reconstruction of bone contour, the prevention of sinus complications—the concept of postoperative “safe sinus” [9,10,44,45,46,47]. Pawar [9] and Strong EB [44] recommend establishing a therapeutic protocol depending on the fracture pattern (involvement of the anterior, posterior, or both walls and bone fragment displacement at each level), obliteration of the nasofrontal canal with secondary drainage disorders (FSOT—frontal sinus outflow tract), and the presence of cerebrospinal fluid (CSF) leak. In this case, the comminuted fracture with more than 2 mm displacement of the bilateral anterior and posterior frontal sinus walls, and the bilateral involvement of FSOT by the fracture lines, as well as of the outer cortex of the frontal squamous portion, required bilateral obliteration or cranialization of the frontal sinuses [9,10,44,45,46,47,48]. Due to the clinical absence of CSF leak up to the time of the intervention, as well as to the absence of intraoperative CSF fistula with callusing evolving in the posterior cortex fracture focus, bilateral cranialization of the frontal sinuses was unnecessary, their obliteration being sufficient in this context [9,10,44,45,46,47,48,49]. Choi et al. [50], in their 10-year retrospective study, indicate a favorable evolution, without complications, in 98% of the cases of posterior frontal sinus wall fractures treated conservatively, even for those with CSF leak, which disappeared spontaneously within 10 days post-trauma. Certainly, in the case of persistent CSF leak, cranialization is required [50]. Chen KT et al. [49] also recommend to wait forfour to seven days before making the decision of cranialization in posterior frontal sinus wall fractures for the same considerations.

Obliteration of the frontal sinus can be achieved with anterior pedicled galeal flap, with autogenous grafts (fat, medullary bone, cortical bone, or muscle), xenografts (bovine bone granules), or various biomaterials (biovitroceramics, polytetrafluoroethylene, calcium phosphate cement, etc.) [8,22,50]. In the present case, we could not use an anterior pedicled galeal flap because of the laceration and incarceration of the epicranial aponeurosis in the comminuted fracture lines of the frontal bone squama, its adequate dissection being practically impossible. At the same time, the patient’s voluminous frontal sinuses would have required a considerable bone amount if obliteration with an autogenous bone graft had been considered. This would have led to higher morbidity at the donor site, under conditions of uncertain bone graft stability over time [8,22,50]. A feasible variant would have been obliteration using biomaterials, which are stable in time and biocompatible, but involve high costs [51]. Literature studies have demonstrated no statistically significant difference in successful frontal sinus obliteration regardless of the material chosen for this purpose [50]. Under these circumstances, it was decided that using cellular adipose tissue autografts taken from the abdomen was the best solution in terms of price-quality ratio for frontal sinus obliteration in the present case. However, there is a risk that the adipose tissue may dissolve, leading to failure of frontal sinus obliteration or even infection; risk quoted below 2% in the specialty literature [50,51].

The contour of the frontal calvaria was reconstructed by open reduction and internal fixation (ORIF) with monocortical miniplates and osteosynthesis screws, similar to the procedure used by other authors [40,41,42,43,44,45,46,47,48,49,50,51,52].

Additionally, open reduction and internal fixation of the left inferior orbital margin fracture were necessary. There are many surgical approaches to the orbital floor, their choice depending on the fracture pattern, the available reconstruction methods, and the surgeon’s preference [53,54,55,56]. Currently, most authors prefer the transconjunctival retroseptal approach due to the invisible extraoral scar and the minimal rate of postoperative complications, the risk of ectropion being practically eliminated [54,55,56]. We chose the infraciliary approach over the transconjunctival approach because of the periorbital edema present at the time of the intervention, which would have limited the access to the entire orbital floor. The infraciliary approach is demanding, requiring an impeccable technique throughout its duration of execution from incision to suture; otherwise, the risk of postoperative ectropion is considerably increased [54,55,56]. In our case, the evolution of the postoperative wound was obviously favorable, without ectropion or lower eyelid mobility disorders.

The present case had a favorable evolution, without postoperative complications, with restored ocular function, disappearance of diplopia, and three-dimensional reconstruction of the midface. This was due to the early surgical intervention; the NOE fracture could be anatomically reduced without the need for medial orbital wall reconstruction, and the two medial canthal insertions were realigned without canthopexy being necessary. Delayed surgery due to inadequate callusing would have probably prevented the perfect reduction of the medial orbital walls by manipulation with the Walsham forceps alone, and additional interventions would have been required, increasing the morbidity of the case.

## 4. Conclusions

Midface NOE fractures associated with frontal sinus wall fractures require correct and early multilateral treatment in order to ensure good functional, cosmetic, and aesthetic results, with three-dimensional reconstruction of bone and soft tissue architecture. Delay of surgery in the absence of other general priority pathologies is not justified; the late treatment of fractures and already established sequelae is much more difficult, and the outcome under these circumstances is uncertain. Although in this case it was not necessary, the neurosurgical evaluation and intervention is of real value in the treatment of this type of trauma, multidisciplinary collaboration being an important aspect of the treatment and the evolution. Constant collaboration with the intensive care physician is required throughout the hospitalization period.
